# Use of a mixed‐methods approach to develop a guidebook with messaging to encourage colorectal cancer screening among Black individuals 45 and older

**DOI:** 10.1002/cam4.6461

**Published:** 2023-08-21

**Authors:** Adjoa Anyane‐Yeboa, Michelle Aubertine, Aisha Parker, Kaitlin Sylvester, Caleb Levell, Emily Bell, Karen M. Emmons, Folasade P. May

**Affiliations:** ^1^ Division of Gastroenterology MGH Boston Massachusetts USA; ^2^ KS&R New York New York USA; ^3^ Ally Research Partners Atlanta Georgia USA; ^4^ American Cancer Society National Colorectal Cancer Roundtable Kennesaw Georgia USA; ^5^ Harvard TH Chan School of Public Health Boston Massachusetts USA; ^6^ Vatche and Tamar Manoukian Division of Digestive Diseases, UCLA Kaiser Permanente Center for Health Equity and Jonsson Comprehensive Cancer Center, University of California Los Angeles Los Angeles California USA

**Keywords:** barriers, colon cancer, colonoscopy, colorectal cancer, screening, stool‐based testing

## Abstract

**Introduction:**

Colorectal cancer (CRC) is the second leading cause of cancer‐related deaths in the United States and disproportionately impacts Black individuals. Here, we describe the mixed‐methods approach used to develop a tailored message guidebook to promote CRC screening among Black individuals in the setting of recently updated screening guidelines.

**Methods:**

This mixed‐methods study included 10 in‐depth qualitative interviews and 490 surveys in a nationally representative sample of unscreened Black individuals age ≥ 45. Messages were developed based on American Cancer Society (ACS) and National Colorectal Cancer Roundtable (NCCRT) research findings, tested among Black individuals using MaxDiff analytic methods, and reviewed by a multi‐sector expert advisory committee of NCCRT members.

**Results:**

The most frequently reported screening barrier in all age groups was self‐reported procrastination (40.0% in age 45–49, 42.8% for age 50–54, 34.2% for age ≥ 55). Reasons for procrastination varied by age and included financial concerns, COVID‐19 concerns, and fear of the test and bowel preparation. Additional screening barriers included lack of symptoms, provider recommendation, and family history of CRC. Most individuals age 45–49 preferred to receive screening information from a healthcare provider (57.5%); however, only 20% reported that a provider had initiated a screening conversation.

**Conclusions:**

We identified age‐specific barriers to CRC screening and tailored messaging to motivate participation among unscreened Black people age ≥ 45. Findings informed the development of the NCCRT and ACS guidebook for organizations and institutions aiming to increase CRC screening participation in Black individuals.

## Graphical abstract

We performed a mixed‐methods study with 10 in‐depth qualitative interviews and 490 surveys in a nationally representative sample of unscreened Black individuals aged 45 and over to identify barriers to screening and effective messaging to prompt screening. The most frequently reported screening barrier in all age groups was self‐reported procrastination, and reasons for procrastination varied by age and included financial concerns, COVID‐19 concerns, and fear of the test and bowel preparation. Effective messages detailed the risk of colorectal cancer (CRC) in Black individuals and highlighted that CRC is preventable with screening.

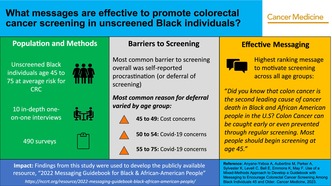

## 1 INTRODUCTION

Colorectal cancer (CRC) is the second leading cause of cancer‐related deaths in the United States and disproportionately impacts Black individuals.^1^ Black individuals have approximately 20% higher incidence and 40% higher mortality from CRC than non‐Hispanic White individuals.^2^ These disparities in incidence and mortality are multifactorial; however, lack of participation in screening is a major contributor.^2^, ^3^, ^4^

Both quantitative and qualitative studies have explored multilevel barriers to screening participation among Black individuals in the United States. However, prior studies have largely focused on one or few settings and on older Black individuals. Given recent guidance from the US Preventive Services Task Force (USPSTF) to initiate CRC screening at age 45,^5^ there is pressing need to understand barriers and facilitators to screening in this younger population of Black individuals as well. The National Colorectal Cancer Roundtable (NCCRT) and American Cancer Society (ACS) have worked to gain a deeper understanding of the barriers to screening across racial/ethnic groups and to provide resources to increase screening rates in underserved populations. They previously completed two comprehensive rounds of market research on reaching unscreened populations and two additional companion pieces to promote screening participation among Hispanic^6^ and Asian individuals.^7^

In this manuscript, we describe the NCCRT and ACS's mixed‐methods approach to identify barriers to CRC screening in a large, nationally representative sample of Black individuals, including a specific focus on those age 45–49. Findings informed the development of the “2022 Messaging Guidebook for Black & African American People: Messages to Motivate for Colorectal Cancer Screening”.^8^ This published resource includes tailored, tested messaging to aid providers, health institutions, organizations, and advocates who aim to increase participation in CRC screening among Black individuals, particularly considering updated CRC screening guidelines.

## 2 METHODS

### 2.1 Study population and recruitment

We conducted a mixed‐methods study that included both in‐depth qualitative interviews and an online survey. For both the interviews and survey, participants were (1) self‐identified Black race, (2) age 45 or older (in 2021), (3) living in the United States, and (4) had no prior CRC screening. The study was reviewed and approved by the Institutional Review Board (IRB) at the Morehouse School of Medicine (1816826–1). Qualitative interviews were performed first to inform the survey instrument development. Interview participants were recruited by the Schlesinger Group,^9^ a national research technology company that uses multiple channels to ensure diverse study participant recruitment, including social media, mobile applications, radio, print media, billboards, affiliates, networks, publishers, and referrals.

For the survey component of study, participants were recruited by Prodege,^10^ a data‐driven marketing and consumer insights platform with a database of individuals who have agreed to receive electronic surveys. Individuals are sent an invitation to complete a survey if they meet specific eligibility criteria defined for the survey. Prodege recruits a diverse sample of survey participants through social media, online and offline advertising, member referrals, and longstanding partnerships with large firms such as United Airlines and Hilton Hotels.

### 2.2 Qualitative interview data collection

The 10 qualitative interviews were conducted between August 12, 2021 and August 17, 2021. An interview guide was created with open‐ended questions and scripted probes. The interview guide was crafted based on similar and previous work done by ACS in this area, which included Black individuals as a subpopulation. The prior work provides some insight into the attitudes, behaviors, and barriers to screening in this population. The guide probed deeper into previously identified barriers and other factors, as well as new topics such as health care access and sociopolitical factors that can influence an individual's ability to be screened. The script was then piloted for appropriateness of content, comprehension, flow, and acceptability with an advisory group comprised of five individuals with backgrounds in public health, policy, advocacy, clinical, market research, health communications, and health equity experts, CRC advocates, and representatives from the NCCRT Public Awareness Strategic Priority Team and Professional Education and Practice Implementation Committee. Modifications were made based on their feedback. One team member (AP) conducted all 10 interviews via a virtual online platform. Each interview lasted approximately 60 min (standard from prior studies), and all interviews that were started were completed. All interviews were transcribed. Individuals received $75 compensation for participation in interviews. Qualitative interviews were used to help inform survey development (below), on which all research findings and conclusions were based.

### 2.3 Survey development and data collection

The American Cancer Society previously conducted studies to assess barriers to CRC screening among unscreened average‐risk individuals.^6^, ^7^, ^11^, ^12^ However, there were few Black individuals included in those studies. The present research borrows from the theoretical basis of prior studies^13^, ^14^ but focuses solely on Black people, obtaining a larger sample size of this demographic in order to report more granular data on barriers and facilitators and to understand differences by age, gender, insurance status, region, and other factors.

The survey instrument was developed, edited, and finalized by the expert advisory group and was based on themes identified from previous qualitative interviews and quantitative research.^6^, ^7^, ^11^, ^12^ The final survey included 76 items; most questions were multiple choice, however six were open‐ended. The survey was organized into 10 modules: (1) demographic information (24 questions); (2) knowledge and awareness about CRC screening (5 questions); (3) discussions with healthcare providers about screening (7 questions); (4) discussions with friends and family about screening (5 questions); (5) current misconceptions and attitudes towards screening (1 question); (6) current sources of information related to CRC screening (1 question); (7) key channels and individuals to receive CRC messaging (3 questions); (8) general health behavior (7 questions); (9) connection to cancer (7 questions); (10) a MaxDiff message testing exercise (12 questions) (16); and (11) celebrity awareness/association (4 questions). The survey was made available via an online platform from October 1, 2021 to October 15, 2021 and took approximately 30 min to complete. Participants received up to $4 as compensation for survey completion.

### 2.4 Data analysis

For qualitative interviews, all data were de‐identified. Interviews were first transcribed by one coder (MA) who manually categorized interview responses, key words, phrases, and quotations into major and minor themes. A second team member (AP) listened to interview recordings, reviewed transcriptions, and confirmed themes. When there were divergent views on themes, the two coders revisited the transcripts together to clarify key findings and come to a consensus. We then consolidated feedback about intervention content, language, and timing to explore participant perspectives of CRC risk, screening tests, and barriers to screening.

We used SPSS (Version 25.0) to perform descriptive statistics for each survey item. We also performed cross‐tabulations to summarize frequencies of responses by age and by family history of CRC.

### 2.5 Guidebook development

After the completion of the qualitative interviews and surveys, the expert advisory group conducted a series of meetings to discuss results and craft potential messages for testing. The advisory group identified four general themes: “CRC is preventable and treatable if caught early”; “CRC is a silent disease”; “family history is important/get screened for your family”; and “there are many screening options.” The expert advisory group used these four themes to create tailored messages over many iterations. Messages then underwent medical and editorial review by an internal team at ACS.

Messages were tested in sets of three using MaxDiff advanced analytic methods.^15^ MaxDiff is a type of conjoint analysis that allows researchers to identify “winning” messages and the degree to which one message is more impactful than another. Messages were tested among the 490 Black individuals who completed the surveys. Study participants were asked to select the message that was “most likely to impact their decision to get screened” and “least likely to impact their decision to get screened.” The messages were framed in this manner to highlight the major motivation(s) for screening, as has been done in prior ACS and NCCRT research.^6^, ^7^ We then calculated share of preference (SOP) true ratios to measure the impression of each message over the others.^16^, ^17^, ^18^ SOP reflects the modeled proportion that a given message would be preferred if all messages in the set were presented. It is the standard output for MaxDiff analysis and not only characterizes the performance of a message (allows researchers to identify top messages) but also helps compare impact between messages. Beyond ACS and NCCRT, conjoint analysis has been used to study patient preferences along the cancer care continuum, including CRC screening,^19^, ^20^ lung cancer screening,^21^ HPV testing,^22^ chemotherapy in breast cancer treatment,^23^ and others. A total of 15 messages were tested for inclusion in the guidebook. The most impactful messages were selected for inclusion in the NCCRT guidebook publication.^8^

## 3 RESULTS

### 3.1 Characteristics of the study participants

The study included 500 unscreened Black individuals (10 qualitative interviews and 490 survey participants) (Table 1). There was no overlap of interview and survey participants. Of the 10 qualitative interview participants, 40.0% were female, and age ranged from 45 to 66 (mean = 52.7, SD = 6.1). Of the 490 survey participants, 53.5% were female, and age ranged from 45 to 82 (mean = 55.3, SD = 7.3). Of the individuals who participated in surveys, 24.5% (*n* = 120) were age 45–49 (Table 1).

**TABLE 1:** Characteristics of study population.

Characteristics, mean (SD) or *N*, %	Survey participants, mean or *N* (%)	Qualitative interview participants, *N* mean or *N* (%)	Total participants, N
Age–mean	55.3	52.7	
45–49	120 (24.5%)	3 (30.0%)	123
50–54	145 (29.6%)	2 (20.0%)	147
55+	225 (45.9%)	5 (50.0%)	230
Race			
Black	490 (100%)	10 (100.0%)	500
Gender			
Female	262 (53.5%)	4 (40.0%)	266
Male	226 (46.1%)	6 (60.0%)	232
Other	2 (0.4%)		
Health insurance type			
Private	153 (37.1%)	6 (60.0%)	159
Medicare	111 (26.9%)	0 (0.0%)	111
State Insurance Program*	119 (28.8%)	1 (10.0%)	120
VA/military	15 (3.6%)	0 (0.0%)	15
Other	38 (9.2%)	1 (10.0%)	39
Uninsured	77 (15.7%)	2 (20.0%)	79
Location Type			
Urban	215 (43.9%)	5 (50.0%)	220
Suburban	211 (43.1%)	4 (40.0%)	215
Rural	62 (12.7%)	1 (10.0%)	63
Not sure	2 (0.4%)	0 (0.0%)	2
Household Income			
Less than 12,000	63 (12.9%)	1 (10.0%)	64
12,000–39,999	187 (38.2%)	2 (20.0%)	189
40,000–59,999	98 (20.0%)	2 (20.0%)	100
60,000–79,000	53 (10.8%)	1 (10.0%)	54
80,000–99,999	22 (4.5%)	1 (10.0%)	23
100,000 or more	49 (10.0%)	3 (30.0%)	52
Prefer not to say	18 (3.7%)	0 (0.0%)	18
Family history of CRC			
Yes	43 (8.8%)	2 (20.0%)	45
No	447 (91.2%)	8 (80.0%)	455
US Region			
Northeast	100 (20.4%)	2 (20.0%)	102
Southeast	187 (38.2%)	4 (40.0%)	191
Southwest	69 (14.1%)	0 (0.0%)	69
Midwest	90 (18.4%)	3 (30.0%)	93
West	44 (9.0%)	1 (10.0%)	45
Marital status		Not asked	
Single/never married	195 (39.8%)		
Married/living partner	174 (35.5%)		
Separated/Divorced/Widowed	118 (24.1%)		
Prefer not to say	3 (0.6%)		
Education			
High school or less	144 (29.4%)	Not asked	
Some college	136 (27.8%)		
Trade or vocational training	21 (4.3%)		
Associates or Bachelors' Degree	137 (27.9%)		
Graduate degree	41 (8.4%)		
Postgraduate Degree	11 (2.2%)		
Employment			
Employed full time	183 (37.3%)	Not asked	
Employed part time	42 (8.6%)		
Retired	77 (15.7%)		
Unemployed or disabled	142 (29.0%)		
Self‐employed	40 (8.2%)		
Student	6 (1.2%)		

* Medicaid, CHIP, etc.

The study population was geographically diverse with the largest percentage from the southeast United States at 38.2%, 43.9% from urban areas, 43.1% from suburban areas, and 12.7% living in rural areas. Most (57.2%) completed some college or less; 29.0% were unemployed or disabled; and 15.7% were retired. Most (84.3%) had health insurance. (Table 1).

### 3.2 Reported barriers to screening by age group

**FIGURE 1:**
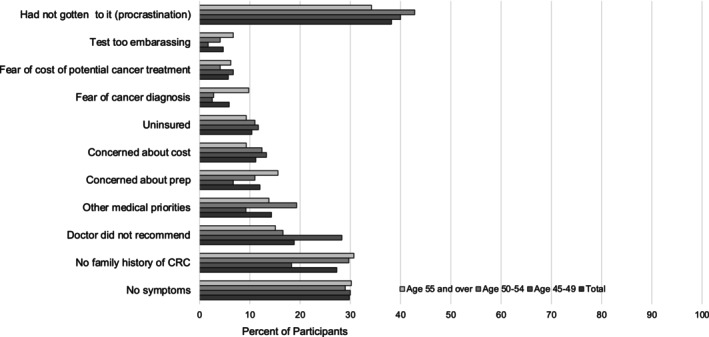
Reasons for not being screened for CRC. Participant reported reasons for being unscreened for colorectal cancer.

The most frequently reported barriers to screening for the population age 45–49 (*n* = 120) were self‐reported procrastination (40.0%), lack of symptoms (30.0%), and lack of provider recommendation (28.3%). Similar findings were observed in the 50–54 age group (*n* = 145) and in the age ≥ 55 group (*n* = 225), with 42.8% and 34.2%, respectively, reporting lack of screening due to procrastination, about one‐third in each group (29.0% for age 50–54 and 30.2% for age ≥ 55) reporting lack of screening due to lack of symptoms, and another third (29.7% for age 50–54 and 30.7% for age ≥ 55) due to lack of family history of CRC **(**Figure 1 **).**

**FIGURE 2:**
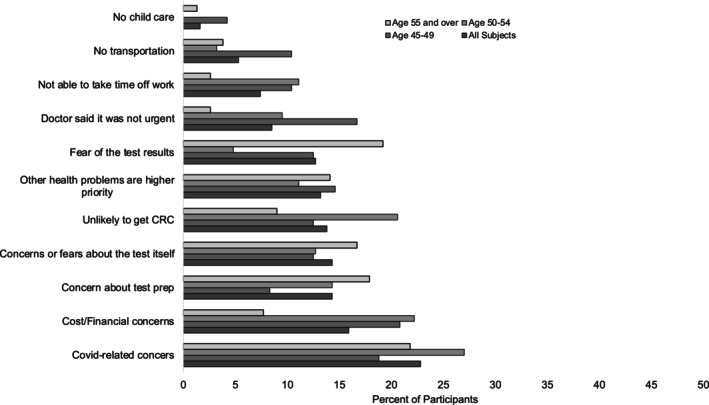
Reasons for procrastination of CRC screening. Participant reported reasons for procrastination of colorectal cancer screening.

Reasons for procrastination varied by age group. In the 45–49 (*n* = 120) age group, the most frequent reasons for self‐reported procrastination were related to cost and financial concerns (20.8%), followed by COVID‐19 pandemic‐related concerns (18.8%), being told by their provider that screening was not urgent (16.7%), and prioritizing other health concerns (14.6%) (Figure 2).

The most common reasons for procrastination in the 50–54 age group (*n* = 145) were related to COVID‐19 pandemic concerns (27.0%). After COVID‐19 concerns, the most common reasons for procrastination in this age group were cost and financial concerns (22.2%) and perception of low CRC risk (20.6%) **(**Figure 2 **).**

In the study subpopulation age ≥ 55 (*n* = 225), COVID‐19 was the most reported reason for procrastination (21.8%), followed by fear of the screening test result (19.2%), concerns about colonoscopy preparation (17.9%), and fears or concerns about the screening test (16.7%) **(**Figure 2 **).**

### 3.3 Conversations about CRC screening

Out of all 490 participants, there were 37.6% of individuals who reported previously discussing CRC screening with friends or family. More than half of participants (51.3%) reported that they felt screening was important after speaking with family and/or friends about screening. However, approximately one in four survey participants (22.9%) walked away from these conversations scared about the process of getting screened for CRC.

**FIGURE 3:**
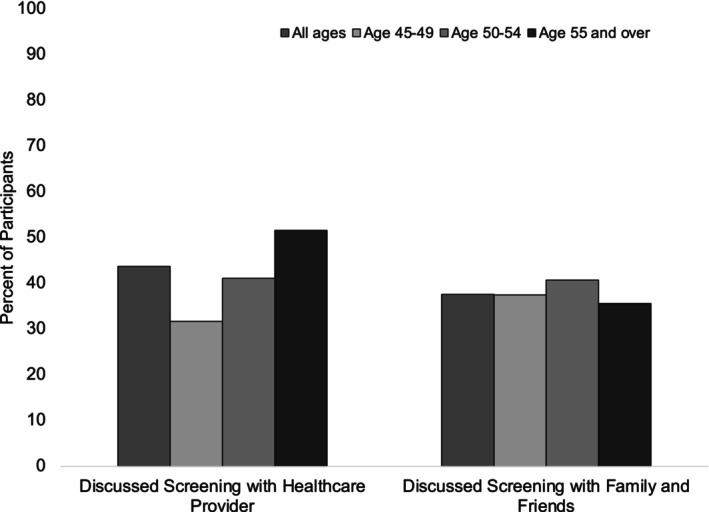
Discussions about CRC Screening by age group. Percentage of participants who had discussions about colorectal cancer screening with healthcare providers and family/friends by age group.

In the group age 45–49 (*n* = 120), only 31.7% of participants age 45–49 reported that their healthcare provider had discussed CRC screening with them **(**Figure 3). These discussions in the 45–49 age group were initiated by the healthcare provider in 63.2% of cases and by the patient in 36.8% of cases. In contrast, in the 50–54 age group (*n* = 145), 41.4% of participants reported that their healthcare provider had discussed screening with them **(**Figure 3 **)**, and 75% of those conversations in those age 50–54 were initiated by the provider. Similarly, in the age ≥ 55 population (*n* = 225), providers had discussed screening with 51.6% of individuals **(**Figure 3), and 83.6% of those conversations were initiated by the provider.

### 3.4 Individuals with a family history of CRC

Of survey participants with a family history of CRC (*n* = 43, 8.8%), 41.9% reported that they planned to be screened eventually but had not been screened yet. Another 18.6% had not been screened because their provider had not recommended it, and 18.6% had not been screened because they were focused on other medical problems. Of the 41.9% with a family history who procrastinated screening, 22.2% reported delays due to fear of the colonoscopy preparation, and 16.7% were unscreened due to fear of the screening test results.

Individuals with a family history of CRC had misconceptions about their own CRC risk with 18.6% somewhat or strongly agreeing that individuals with a healthy lifestyle do not need to be screened, and 7.0% believing that CRC was only possible in men. Of those with a family history of CRC (*n* = 43), 62.8% reported that they had discussed CRC screening with family and/or friends. Only 48.4% reported that they planned to undergo screening in the near future.

### 3.5 Motivating Messages

The highest ranked message to motivate screening was “Did you know that colon cancer is the second leading cause of cancer death in Black and African American people in the United States? Colon Cancer can be caught early or even prevented through regular screening. Most people should begin screening at age 45”. At 15.7%, this message had the highest SOP and ranked in the top three among 49.6% of all participants. It was also the top message across all age groups, including Black individuals age 45–49 who are newly eligible for screening: 17% for participants age 45–49, 15.6% for age 50–54 and 15.1% for age ≥ 55. (Table 2).

**TABLE 2:** Top screening messages by age group.

Category	Message content
Top messages	
All ages	“Did you know that colon cancer is the second leading cause of cancer death in Black and African American People in the United States? Colon Cancer can be caught early or even prevented through regular screening.”
	2 “Colon cancer is often a silent disease. Usually there are no symptoms. That's why getting screened is so important. It can help prevent colon cancer—or catch it early when it is easiest to treat. Most people should begin screening at age 45. It can help prevent colon cancer–or catch it early when it is easiest to treat. Most people should begin screening at age 45.”
Age 45–49	“Did you know that colon cancer is the second leading cause of cancer death in Black and African American People in the United States? Colon cancer can be caught early or even prevented through regular screening.”
	2 “Colon cancer is often a silent disease. Usually there are no symptoms. That's why getting screened is so important. It can help prevent colon cancer—or catch it early when it is easiest to treat. Most people should begin screening at age 45. It can help prevent colon cancer—or catch it early when it is easiest to treat. Most people should begin screening at age 45.”
Age 50–54	“Did you know that colon cancer is the second leading cause of cancer death in Black and African American People in the United States? Colon cancer can be caught early or even prevented through regular screening.”
	2 “Colon cancer still happens more often in African American, but progress is being made. Fewer African American people develop or die from colorectal cancer as compared to just a few years ago, thanks to more African Americans taking part in screening, now starting at age 45.”
Age 55 and over	“Did you know that colon cancer is the second leading cause of cancer death in Black and African American People in the United States? Colon cancer can be caught early or even prevented through regular screening.”
	2 “Colon cancer is often a silent disease. Usually there are no symptoms. That's why getting screened is so important. It can help prevent colon cancer—or catch it early when it is easiest to treat. Most people should begin screening at age 45. It can help prevent colon cancer—or catch it early when it is easiest to treat. Most people should begin screening at age 45.”

Another highly preferred message was, “Colon cancer is often a silent disease. Usually there are no symptoms. That's why getting screened is important. It can help prevent colon cancer–or catch it early when it is easiest to treat. Most people should begin screening at age 45.” This message had the next highest SOP at 9.8% and ranked in the top 3 for 34.9% of participants. The published NCCRT guidebook provides the most impactful messages and additional information and guidance about CRC screening in Black individuals.^8^

### 3.6 Most effective channels to receive CRC screening information

The most perferred channels to receive CRC screening information overall were through discussion with a doctor or healthcare provider (55.5%), a handout or a poster in a provider's office (31.6%), or via email (29.2%). For individuals age 45–49, preferred channels to receive screening were similar. Most individuals (89.4%) found it most impactful to have an everyday person that they relate to speaking about their experience with CRC. Findings were similar for the subset age 45–49.

## 4 DISCUSSION

In 2021, the USPSTF updated their CRC screening guidelines, recommending CRC screening for individuals age 45–49 for the first time.^24^ Motivated by the new CRC screening guidelines and persistent Black‐White disparities in CRC incidence and mortality, we performed a mixed‐methods study to identify barriers to screening and develop effective messages to prompt screening in unscreened Black individuals. We found that the most common barrier to screening was self‐reported procrastination in all age groups, followed by lack of symptoms and lack of family history of CRC. Those who reported procrastination were willing to be screened but had not prioritized screening. The majority of individuals age 45–49 had not discussed screening with their healthcare provider, and were less likely to report that providers initiated conversations about screening than participants age 50 and above.

The use of culturally tailored information to prompt CRC screening has been previously studied. One study noted that culturally tailored messaging increased receptiveness to CRC screening and reduced anticipatory racism stress among unscreened Black individuals age 50–75.^25^ In another study, culturally tailored CRC education was noted to help to overcome barriers to screening in 48% of study participants and led to 80% of participants making the decision to undergo CRC screening.^26^

Prior work has provided insight into methods and channels to advocate for screening in Black individuals. One study reported that Black patients preferred CRC screening education from their doctor, family members, survivors they knew, and advocacy organizations.^27^ In our study, we found that the preferred channel for CRC screening information was a doctor or other healthcare provider, followed by a handout or poster in a doctor's office. Interestingly, the preferred channel to receive healthcare information was from a doctor or healthcare provider even among participants who were uninsured, unemployed, or did not have a primary care provider. Our study also provides a ranking of effective messages and channels to receive CRC messaging by age group, including the newly eligible age 45–49 population.

The NCCRT and ACS previously developed messaging guidebooks as resources to prompt screening in Asian and Hispanic/Latino individuals. Prior guidebooks provided information on perceptions around screening, recommendations to reach each group, and culturally tailored messaging to prompt screening based on common values in each specific population. Similarly, the results from the current study were used to develop the “2022 Messaging Guidebook for Black & African American People: Messages to Motivate for Colorectal Cancer Screening”.^8^ This resource is a publicly accessible tool to provide culturally tailored approaches and education for healthcare organizations, nonprofit organizations, providers, policy makers and CRC advocates.

Our study is not without limitations. First, we used a mixed‐methods approach that included in‐depth interviews and surveys that are subject to recall bias. However, findings from in‐depth interviews were supported by survey responses, which is reassuring. A second limitation is that our study is not generalizable to all Black individuals in the United States. Our study only included Black unscreened individuals who had access to the internet, where much of patient recruitment occurred. In addition, relatively few participants were employed full‐time (37.3%), many lived in the Southeast United States (38.2%), the majority were insured and unscreened, and most were from urban or suburban areas. Of note, Black individuals in the United States are concentrated in the Southeast and in urban and suburban areas. Nonetheless, our findings should not be extrapolated to all Black people. Third, participants included in the study were recruited from companies with databases of individuals who are interested in participating in surveys and may underestimate the views of individuals who are less motivated. These recruitment strategies did allow us to include a large study population with representation from around the country, however, which was critical to inform the guidebook. Fourth, we did not have information that would have been helpful for further analysis, including the total number of individuals approached for surveys and interviews, specific reasons for cost concerns among procrastinators (e.g., insurance co‐pay, time off work, out of pocket costs), and information on screening participation after data collection. Last, while the guidebook provides messaging intended to prompt Black individuals to get screened for CRC, its ability to actually increase CRC screening participation is currently unknown. Future studies will utilize these messages as an intervention and evaluate their impact on screening test use in Black individuals. Our hope is that, if effective, the guidebook can then be used on a large scale to develop broad messaging campaigns to reach unscreened Black individuals, or on a smaller scale in one‐on‐one patient–provider interactions.

Despite these limitations, our study had several strengths. We provide new insight into the barriers and facilitators of CRC screening among unscreened Black individuals with the unique perspectives of the newly eligible group age 45–49. Specifically, we noted fewer provider‐initiated conversations about CRC screening with individuals age 45–49. This finding is a call to action for providers to address CRC screening at a younger age and provides a target for future screening interventions. In addition, the work represents a nationally representative sample of Black individuals with modest representation of different age groups, both sex groups, and urban and rural populations. In addition, we conducted a mixed method study which provides granular perspectives from qualitative data in addition to quantitative data from a larger group of participants to add generalizability and external validity to findings.

In conclusion, we present the development of a messaging guidebook available to organizations, institutions, and communities working to address CRC screening disparities and increase screening participation among Black individuals in all parts of the country. By developing tailored interventions to address CRC screening disparities in Black people, we can raise awareness about the importance of screening, overcome mutable barriers to screening participation, and help improve CRC outcomes in the communities that see the most devastating impact of this largely preventable disease.

## AUTHOR CONTRIBUTIONS

**Adjoa Anyane‐Yeboa:** Conceptualization (equal); visualization (equal); writing – original draft (lead); writing – review and editing (lead). **Michelle Aubertine:** Formal analysis (equal); investigation (equal); methodology (equal); software (equal); validation (equal); writing – review and editing (equal). **Aisha Parker:** Formal analysis (equal); investigation (equal); writing – review and editing (equal). **Kaitlin Sylvester:** Conceptualization (equal); methodology (equal); project administration (equal); writing – review and editing (equal). **Caleb Levell:** Conceptualization (equal); methodology (equal); project administration (equal); writing – review and editing (equal). **Emily Bell:** Conceptualization (equal); methodology (equal); project administration (equal); writing – review and editing (equal). **Karen M. Emmons:** Visualization (equal); writing – review and editing (equal). **Folasade P. May:** Conceptualization (equal); supervision (lead); visualization (equal); writing – original draft (equal); writing – review and editing (equal).

## FUNDING INFORMATION

AAY and KEM receive grant support from the National Cancer Institute at the National Institutes of Health (grant number P50CA244433). AAY receives grant support from the Trefler Foundation via MGH Cancer Center. FPM is supported by the UCLA Jonsson Comprehensive Cancer Center and the Eli and Edythe Broad Center of Regenerative Medicine and Stem Cell Research Ablon Scholars Program.

## DISCLOSURES

AAY receives research funding from Pfizer Inc and Exact Sciences, and consulting fees from Janssen. FM receives funding support from Exact Sciences and is a consultant for Freenome, Takeda, Johnson and Johnson, and Medtronic. Quest Diagnostics and Elevance Foundation sponsored this work but did not dictate study design, results, or findings.

## ETHICS STATEMENT

The study was reviewed and approved by the Institutional Review Board (IRB) at the Morehouse School of Medicine (1816826‐1).

## DISCLAIMER

This manuscript is the result of the author's own work and has not been published elsewhere.

## Supporting information


Appendix S1:

## Data Availability

The data that support the findings of this study are available from the American Cancer Society and National Colorectal Cancer Roundtable. Restrictions apply to the availability of these data, which were used under license for this study.
