# Supplementary material for: Use of a mixed‐methods approach to develop a guidebook with messaging to encourage colorectal cancer screening among Black individuals 45 and older
Source: Cancer Med. 2023 Aug 21;12(18):19047–56. doi: 10.1002/cam4.6461 (PMC10557828; doi:10.1002/cam4.6461)
Supplement: Supplementary file 1 — Appendix S1: [file CAM4-12-19047-s001.pdf]

APPENDIX 1

<u>Full list of tested messages</u>

Screening theme/Tonality	Lead with Fact	Emotional Appeal (e.g., cheerleader, family focus, other)	Personal story	Miscellaneous
**Preventable and treatable if caught early**	Colon cancer is often preventable and likely to be successfully treated if caught early. Take control of your health and talk to your doctor about when getting screened is right for you.		Carl feels great about his health. He eats right and exercises regularly. And he received good news after his last colon cancer screening. The doctor found and removed growths (polyps) early before they had a chance to become cancer. You can get screened to as a way to take care of yourself. Take control of your health and talk to your doctor today.	Being active and eating right are important for your health, and so is getting screened for cancer. Getting screened for colon cancer can help prevent it or catch it early when it is most treatable. Take control of your health and talk to your doctor today.
**Silent disease**	Colon cancer is often a silent disease. Usually there are no symptoms. That's why getting screened it so important. It can help prevent colon cancer — or catch it early when it is easiest to treat. Most people should begin screening at age 45.	Right now, you could have a polyp, a small growth in your colon or rectum. Right now, your polyp may be harmless, but over time it could develop into colorectal cancer. Right now, through regular screening, you have the power to find and remove precancerous polyps and prevent colorectal cancer. Call your doctor and take control of your health!		
**Family – family history and/or "do it for your family"**	One in four people with colon cancer have a family history of the disease. Family history puts you at an increased risk. Don't delay your care. Protect yourself, so you can be there for your family. Get screened.		My mom was screened for colon cancer and caught it early when it was more treatable. I talked to my doctor and learned I should get screened more often so now I follow the schedule that's right for me. I want to be here for my family like my mom still is for me.	My mom was diagnosed with colon cancer at age 55. I now know I'm at a higher risk for the disease and because of this, my doctor recommend I started screening at age 40. I get regular exams and watch for symptoms. I want to be here for my family.
**Screening options**	Colon cancer screening can save your life. And for most people, there's more than one screening option available to meet your needs, including some that can be done in the privacy of your own	Did you know screening is used in people who don't have any symptoms? It is safe, can find colon cancer early, and can even help prevent it too. There are several		A colonoscopy isn't the only option for colorectal cancer screening. There are simple, affordable options, including tests that can be done at home. Talk to your doctor

	home. Most people should begin screening at age 45. Talk to your doctor today about which option is right for you.	screening tests available, some of which can be done at home. Talk to your doctor about which one is right for you.		about which option is right for you. Ask which tests are covered by your health insurance.
**MISC and/or includes statistics related to Black and African American communities**	Did you know that colon cancer is the second leading cause of cancer death in Black and African American people in the United States? Colon cancer can be caught early or even prevented through regular screening. Most people should begin screening at age 45.	Colon cancer still happens more often in African Americans, but progress is being made. Fewer African American develop or die from colorectal cancer as compared to just a few years ago, thanks to more African Americans taking part in screening, now starting at age 45.	Al's buddy, Joe, is a colon cancer survivor. Joe was only 50 when he was diagnosed. Al just turned 45 and feels healthy, but so did Joe before he got screened. Al now knows colon cancer screening is a way to take care of himself, so he plans to talk to his doctor about getting screened as soon as possible.	Did you know that most African Americans are screened for colon cancer? Most African Americans believe that others should be screened too. Ask your friends and family and they'll probably agree – starting screening at age 45 is the right thing to do.

Message Testing For Black & African American Unscreened People - Quantitative Survey

Survey Notes

- Target Audience: **500** survey completes amongst unscreened Black/African American respondents ages 45+ years old.
    - Live in the US
- Survey length: 15 minutes
- Unbranded

Thank you for taking the time to respond to our survey! Your feedback is greatly appreciated! Please be assured that all of your responses are confidential and will be used for research purposes only.

As you move through the survey, please use only the "Previous Screen <" or the "Next Screen >" button at the bottom of the page.

This survey will take about **15** minutes.

Screener

S1. Are you…?

1. Male
2. Female
3. Transgender male to female
4. Transgender female to male
5. Non-binary
6. Prefer not to answer

S2. What year were you born?

[ ]

PROGRAMMER COMPUTE AGE AND THANK & TERMINATE IF < 45 years old

[Programming Note: Show S6 and S7 on same screen]

S6. Are you of Spanish, Hispanic, or Latino origin or descent?

1. Yes

2. No

S7. Do you consider yourself …?

1. White
2. Black or African American
3. Asian or Pacific Islander
4. Native American or Alaskan native
5. Biracial or multiracial
6. Other

IF S7a=2 ASK:
S7b. Can you be more specific on the race you identify with? For example, African American, Jamaican, Haitian, Ethiopian, Somali, etc. (open-end)

S3. Are you, or is any member of your family or household, employed in any of the following lines of work? *(Select all that apply)***[RANDOMIZE]**

1. Media or news broadcasting
2. A doctor's office, hospital, or urgent care facility
3. Cancer related non-profit organization
4. None of these **[ANCHOR, EXCLUSIVE]**

IF S7<> 2 THANK & TERMINATE
IF S3=1,2, or 3 THANK & TERMINATE

S4. Please select the state in which you live. **(State drop down menu, code 1-51)**

S5. Please enter your 5-digit zip code. (**FORCE VALID US ZIP, FILL IN THE BLANK)**

Q1 For each of the following medical procedures below, please indicate whether or not you have **EVER** had it done. (Select one for each) **[RANDOMIZE]**

1. Yes, I've done this in the past 2. No, I've never done this

1. Had a colon cancer screening test (i.e. colonoscopy, stool sample test, Cologuard test, CT Colonography, flexible sigmoidoscopy, Septin9 methylated blood test, capsule endoscopy)
2. Had a mammogram
3. Had a prostate cancer screening test (i.e. PSA blood test)
4. Received a COVID-19 vaccine
5. Received a flu shot
6. Had LASIK eye surgery

7. Had cataract surgery
8. Had a knee replacement
9. Had a hip replacement

IF Q1=1 ASK:

Q1a. We'd like to understand what motivated you to get screened for colon cancer. Please complete the following statement: ***I decided to get screened for colon or colorectal cancer after…*** (Select all that apply) **[RANDOMIZE]**

1. Experiencing stomach or intestinal problems, such as blood in the stool, stomach aches, etc.
2. Talking with a friend or family member
3. Finding out I have a family history of colon cancer
4. Talking with a leader in my faith/church
5. Talking with my doctor or other health care provider
6. Getting information from my health plan or insurance company
7. Getting information from my employer
8. Hearing or seeing information on the radio or television (e.g. advertisement, news story, public service announcement)
9. Seeing a news story or advertisement in print (e.g. brochure, newspaper article)
10. Seeing information on a billboard
11. Seeing something on social media
12. Other **[ANCHOR]**

IF Q1=1 (HAVE BEEN SCREENED FOR CRC) THANK & TERMINATE

Colon Cancer Screening

Now we'd like to talk to you about a specific health related topic.

Cancer is a disease in which cells in the body grow out of control. Colorectal cancer is cancer that starts in the colon or rectum. The colon is the large intestine or large bowel. The rectum is the last few inches of the colon. Sometimes colorectal cancer is called colon cancer, for short. For the remainder of the survey we will use the term colon cancer, but please know we are referring to both colon and colorectal cancer.

Q2b. What do you think the chances of survival are for someone diagnosed with colon cancer? (Select one)

1. **Very high** – most people will survive
2. **Somewhat high** – the majority of people will survive
3. **Moderate** – about half of the people diagnosed will survive
4. **Somewhat low** Some people will survive, but more people will die from it
5. **Extremely low** – very few people will survive

Q3. What comes to mind when you think of screening tests for colon cancer? (Open-end)

[DO NOT ALLOW RESPONDENTS TO GO BACK AFTER Q3]

Awareness of screening tests

S10a. How familiar are you with each of these tests that are used to look for colon cancer? **[RANDOMIZE]**

1. Never Heard Of This Test	2. Heard Of This Test

1. **Stool test, also called a Fecal Occult Blood Test (FOBT) or Fecal Immunochemical Test (FIT):** This is a test that you do at home; you have to collect one or more stool samples to send back to the doctor or a lab to be checked for blood in the stool.

2. **Colonoscopy:** For this test, you must clean your colon leading up to the exam by taking laxatives (pills) or drinking a mixture including a laxative (or both). During the exam a long narrow tube is inserted into the rectum to look at **the inside of the entire colon**. You are usually given medication to help you relax or go to sleep, and you must have someone else drive you home afterward.
3. **FIT-DNA test or Stool DNA Test (known by the brand name, "Cologuard"):** This test looks for DNA changes in the cells of a stool sample. You use a kit at home to collect an entire sample of stool and mail it to a lab. This test looks for DNA changes associated with colon polyps and colon cancer.
4. **CT Colonography (CTC):** CT Colonography is an exam during which your colon is examined by a CT scanner. Like colonoscopy, you need to drink a laxative to clean your colon. A CT scan uses x-rays, but instead of taking one picture, like a regular x-ray, a CT scanner takes many pictures as it rotates around you while you lie on a table. A small, flexible tube is also put in the rectum for this test to fill the colon and rectum with air. You **do not** need medication to help you relax or sleep with this test.

Discussions with health care providers

Q7.Have you and your doctor or health care provider ever talked about anything regarding colon cancer screening tests (e.g. stool tests, colonoscopy, etc.)?

1. Yes
2. No
3. I do not recall

If Q7=1 (YES) ASK:

Q8. From what you can remember, who started the initial conversation about colon cancer screening? *(Select one)*

1. I did
2. My health care provider/doctor did

3. Someone else did (please specify _________) [ANCHOR]
4. I do not recall [ANCHOR]

IF Q7=1 (YES), ASK

Q7a. To the best of your knowledge, how old were you when you had your first conversation with your doctor or health care provider about colon cancer screening? **(Insert drop down list for age)**

IF Q7=1 (YES), ASK:

Q9a. If your health care provider or doctor has ever talked to you about screening for colon cancer, was it because: *(Select all that apply)* **[RANDOMIZE]**

1. He or she said I was due for screening at the time of the discussion
2. He or she wanted to prepare me, since I would be due for screening in the coming years
3. He or she was discussing whether or not I had a family history of colon cancer
4. He or she was discussing whether or not I had a family history of ANY cancer [ANCHOR BELOW OPTION 3}
5. He or she noted symptoms/signs and wanted to rule out colon cancer with testing
6. Other (please specify ____) **[ANCHOR]**

IF Q7=1 (YES), ASK:

Q9b. Which of the following best describes the importance your doctor placed on getting screened for colon cancer? *(Select one)* **[RANDOMIZE]**

1. They made it seem urgent/something I should do as soon as possible
2. They noted it is important to do eventually, but it didn't seem urgent
3. They just noted I should get screened but did not provide a sense of how important it is

Q10. Which of the following, if any, has a doctor or health care provider discussed with you about colon cancer? *(Select all that apply)* **[RANDOMIZE]**

1. Whether or not I had a family history of colon cancer
2. Informed me those with a family history are at an increased risk of getting colon cancer
3. Lifestyle changes I could make to reduce my risk of colon cancer (e.g., reduce red meat consumption, eat more fiber, stop smoking, reduce alcohol intake, exercise more, etc.)
4. Symptoms/signs of colon cancer
5. Other (please specify _____) **[ANCHOR]**
6. None, I have never discussed colon cancer with a doctor or health care provider **[ANCHOR, EXCLUSIVE]**

Q10b. How comfortable are you discussing the following topics with a doctor or health care provider? **[RANDOMIZE]**

Not at all comfortable	Somewhat uncomfortable	Neither comfortable nor uncomfortable	Somewhat Comfortable	Very Comfortable
1	2	3	4	5

1. Colon cancer screening
2. Cancer in general
3. Your bowel habits
4. Rectal discomfort
5. Stomach/intestinal discomfort

Discussions with family & friends

Q11. Have you ever heard family or friends talk about colon cancer screening? *(Select all that apply)*

1. Yes, they've talked directly to me about it
2. Yes, I've overheard them discussing it with others
3. **3.** No **[EXCLUSIVE**

If Q11=1 or 2 ASK:

Q12. What specifically do you recall about those conversations about colon cancer screening? *(Select all that apply)* **[RANDOMIZE]**

1. If we've had colon cancer screening tests yet
2. Reasons why testing for colon cancer may be important
3. The age when we are supposed to start colon cancer screening
4. That we're getting older and have hit the age to start colon cancer screening
5. Symptoms of colon cancer
6. Whether screening can prevent colon cancer
7. Whether I or they have a family history of colon cancer

8. The different types of tests that can be used to screen for colon cancer
9. The next steps for getting screened for colon cancer (e.g. how to make an appointment, test procedures, directions for prepping for the test, etc.)
10. How much colon cancer screening costs
11. What the prep for the test entails
12. What the actual test entails
13. Something else (please specify ______) **[ANCHOR]**

Impact of discussions with friends/family

If Q11=1 or 2 ASK:

Q13. How did the discussions with family and friends about colon cancer screening make you feel? *(Select all that apply)* **[RANDOMIZE]**

1. Made me feel that colon cancer screening is important
2. Made me scared to think about the actual process of getting a screening test
3. Made me worried that I would get colon cancer
4. Made me never want to get screened for colon cancer
5. Felt uncomfortable talking about the topic
6. Made me feel more comfortable about getting screened some day
7. Made me want to learn more about colon cancer or colon cancer screening
8. Other (please specify ____) **[ANCHOR]**
9. Didn't really impact me in any way **[ANCHOR]**

Q14. Which of the following describes why you have not had a colon cancer screening test yet? (Select all that apply) **[RANDOMIZE]**

1. Don't have symptoms
2. Heard the prep was unpleasant
3. No family history of Colon Cancer
4. Can't afford the out of pocket costs
5. Test is too embarrassing
6. Doctor didn't tell me to get tested
7. Don't have insurance
8. Don't want to know if I have cancer
9. If they find cancer, I can't afford treatment
10. Focused on other medical problems
11. Other (please specify ___) **[ANCHOR]**
12. Will do it eventually/just haven't yet **[ANCHOR]**

IF Q14=12 ASK:

Q15. You mentioned you will get screened eventually but haven't yet. Which of the following reasons, if any, describes why you've put off getting screened? (Select all that apply) **[RANDOMIZE]**

1. Don't think you are likely to get colon cancer
2. Concerns about the test prep
3. Concerns or fears about the test itself
4. Fear of the test results
5. Other health concerns are a higher priority for you
6. Cost/financial concerns

7. Doctor told you it wasn't urgent
8. Can't take time off of work
9. Don't have transportation / don't have anyone to take you
10. Don't have child care for your kids or grandkids
11. COVID related concerns
12. Other (please specify ___) **[ANCHOR]**

Identify the current misconceptions and attitudes towards CRC screening

Q17. Please rate your level of agreement with the following statements related to colon cancer screening. **[RANDOMIZE]**

Strongly Disagree	Somewhat Disagree	Neither Agree nor Disagree	Somewhat Agree	Strongly Agree
1	2	3	4	5

1. I don't have a family history of colon cancer so I don't think it's important for me to ever get screened
2. Only those who have symptoms should be screened for colon cancer
3. You don't need to get screened for colon cancer if you eat right and exercise
4. Colon cancer screening is just for men
5. I'm scared of the actual procedure to screen for colon cancer
6. Talking about colon cancer or colon cancer screening is embarrassing
7. I think it's important to increase awareness of colon cancer and colon cancer screening
8. Colon cancer is rare

Current sources of information related to CRC

Q6. Where have you ever seen or heard information about colon cancer screening tests? *(Select all that apply)* **[RANDOMIZE]**

1. Handout or poster in a doctor's office, clinic, or hospital
2. Discussion with a doctor or other health care provider
3. Message or information from my health plan/insurance company
4. Message or information from my employer
5. Discussion with a friend or family member
6. Discussion with my pharmacist
7. Speaker at a meeting or other event
8. Advertisement (e.g. television, radio, Internet, magazine, billboard, etc.)
9. News report or story (e.g. television, radio, newspaper, magazine, Internet, etc.)
10. Website

11. Social Media advertisement (e.g. an ad on Facebook, Twitter, or Instagram)
12. Social Media post by friend or family member
13. Some other place (please specify ______)**[ANCHOR]**
14. Have not seen or heard any information about colon cancer screening tests **[ANCHOR]**

Celebrity awareness/association

Q11a. Can you think of any celebrities, influencers, or public figures you know of that have had colon cancer or have spoken out about getting screened for colon cancer?

1. Yes
2. No

Q11b. Which of the following is more impactful to you (i.e. what would make you more likely to consider getting screened)? (*Select one)* [RANDOMIZE]

1. An everyday person you relate to (same race, age, gender) speaking about their experience with colon cancer
2. A celebrity talking about their experience with colon cancer

Q11c. And when thinking about celebrities, what is more impactful to you - seeing a celebrity like Will Smith getting screened for colon cancer or hearing of a celebrity like Chadwick Boseman dying of colon cancer? (*Select one)* [RANDOMIZE]

1. Seeing a celebrity get screened for colon cancer
2. Hearing of celebrity dying of colon cancer

Q18. How likely are you to make an appointment for colon cancer screening in the next 6 months? Please use a scale of 1 to 10 where 1 means, “Not at all likely” and 10 means, “Extremely likely”.

Not At All Likely									Extremely Likely
1	2	3	4	5	6	7	8	9	10

MaxDiff Message Testing Exercise
Next, you will be shown lists of potential messages that could be used in informational materials and advertisements to encourage people to be screened for colon cancer. You will see **3** messages at a time, across 15 screens. Some of these messages may be shown more than once.

When thinking about messages that may encourage you to get screened for colon cancer, please choose the one message that is **most** likely to impact your decision to get screened and the one message that is **least** likely to impact your decision to get screened.

Programming Note: Respondents will see 15 cards with 3 messages per card. Randomize card order.

When thinking about messages that may encourage you to get screened for colon cancer, please choose the one message that is most likely to impact your decision to get screened and the one message that is least likely to impact your decision to get screened.		
	MOST likely to impact your decision to get screened **SELECT ONE**	LEAST likely to impact your decision to get screened **SELECT ONE**
XX	○	○
XX	○	○
XX	○	○

After each MaxDiff card, insert the following question:
ONLY ASK QUESTION FOR EACH MESSAGE ONCE; IF MESSAGE IS SELECTED AS 'MOST LIKELY' MORE THAN ONCE, SKIP AND MOVE ONTO NEXT CARD.

M1 – M9. Given the message you just selected as the one that would '**most** likely impact your decision to get screened,' **[INSERT 'MOST LIKELY' MESSAGE SELECTED],** how likely are you to get screened for colon cancer in the next 6 months? Please use a scale of 1 to 10 where 1 means, "Not at all likely" and 10 means, "Extremely likely".

Not At All Likely									Extremely Likely
1	2	3	4	5	6	7	8	9	10

MaxDiff anchor question

M10. Which of the following messages would make you more likely to get screened for colon cancer in the next 6 months? **[RANDOMIZE]** ***Please select all that apply.***

**1.**	Colon cancer is often preventable and likely to be successfully treated if caught early. Take control of your health and talk to your doctor about when getting screened is right for you.	
**2.**	Carl feels great about his health. He eats right and exercises regularly. And he received good news after his last colon cancer screening. The doctor found and removed growths (polyps) early before they had a chance to become cancer. You can get screened too as a way to take care of yourself. Take control of your health and talk to your doctor today.	
**3.**	Being active and eating right are important for your health, and so is getting screened for cancer. Getting screened for colon cancer can help prevent it or catch it early when it is most treatable. Take control of your health and talk to your doctor today.	
**4.**	Colon cancer is often a silent disease. Usually there are no symptoms. That's why getting screened is so important. It can help prevent colon cancer — or catch it early when it is easiest to treat. Most people should begin screening at age 45.	
**5.**	Right now, you could have a polyp, a small growth in your colon or rectum. Right now, your polyp may be harmless, but over time it could develop into colorectal cancer. Right now, through regular screening, you have the power to find and remove precancerous polyps and prevent colorectal cancer. Call your doctor and take control of your health!	
**6.**	One in four people with colon cancer have a family history of the disease. Family history puts you at an increased risk. Don't delay your care. Protect yourself, so you can be there for your family. Get screened.	
**7.**	My mom was screened for colon cancer and caught it early when it was more treatable. I talked to my doctor and learned I should get screened more often so now I follow the schedule that's right for me. I want to be here for my family like my mom still is for me.	
**8.**	My mom was diagnosed with colon cancer at age 55. I now know I'm at a higher risk for the disease and because of this, my doctor recommends I started screening at age 40. I get regular exams and watch for symptoms. I want to be here for my family.	
**9.**	Colon cancer screening can save your life. And for most people, there's more than one screening option available to meet your needs, including some that can be done in the privacy of your own home. Most people should begin screening at age 45. Talk to your doctor today about which option is right for you.	
**10.**	Did you know screening is done by people who don't have any symptoms? It is safe, can find colon cancer early, and can even help prevent it too. There are several screening tests available, some of which can be done at home. Talk to your doctor about which one is right for you.	
**11.**	A colonoscopy isn't the only option for colorectal cancer screening. There are simple, affordable options, including tests that can be done at home. Talk to your doctor about which option is right for you. Ask which tests are covered by your health insurance.	

**12.**	Did you know that colon cancer is the second leading cause of cancer death in Black and African American people in the United States? Colon cancer can be caught early or even prevented through regular screening. Most people should begin screening at age 45.	
**13.**	Colon cancer still happens more often in African Americans, but progress is being made. Fewer African American people develop or die from colorectal cancer as compared to just a few years ago, thanks to more African Americans taking part in screening, now starting at age 45.	
**14.**	Al's buddy, Joe, is a colon cancer survivor. Joe was only 50 when he was diagnosed. Al just turned 45 and feels healthy, but so did Joe before he got screened. Al now knows colon cancer screening is a way to take care of himself, so he plans to talk to his doctor about getting screened as soon as possible.	
**15.**	Did you know that most African American people are screened for colon cancer? Most African Americans believe that others should be screened too. Ask your friends and family and they'll probably agree – starting screening at age 45 is the right thing to do.	
**99.**	**None of the above**	

IF MORE THAN ONE MESSAGE SELECTED IN M10, ASK M11 (SKIP IF NONE OF THE ABOVE)
IF A SINGLE MESSAGE IS SELECTED IN M10, AUTOPOPULATE M11 WITH THAT MESSAGE
M11. Which message would make you **most** likely to get screened? ***Please select one.***

SHOW ONLY MESSAGES SELECTED IN M10

M12. What about the message you selected makes you **most** likely to get screened? **(OPEN END)**

Channels & Sources for Health Care Information

Identify key channels for CRC messaging

Q20. Where or how would you like to receive information related to colon cancer screening? *(Select all that apply)* **[RANDOMIZE]**

1. Handout or poster in a doctor's office, clinic, or hospital
2. Discussion with a doctor or other health care provider
3. Discussion with a friend or family member
4. Discussion with a leader from my faith-based institution
5. Advertisement (e.g. television, radio, Internet, magazine, billboard, etc.)
6. Speaker at a meeting or other event
7. News report or story (e.g. television, radio, newspaper, magazine, Internet, etc.)
8. Website
9. Text messages
10. Social media, such as Facebook, TikTok, Reddit
11. Email
12. Patient health online portal
13. Other (please specify ____) **[ANCHOR]**

Identify key people to deliver messages

Q20a. For each of the sources listed below, please rate how much you trust health care information they may give you. *(Select one for each)* **[RANDOMIZE]**

Do not trust at all	Somewhat Distrust	Neither Trust nor Distrust	Somewhat Trust	Trust completely
1	2	3	4	5

1. Doctors
2. Other medical professionals (nurse, nurse practitioner, physician's assistant) **[ALWAYS ANCHOR BELOW OPTION 1 – DOCTORS]**
3. My health insurance plan
4. My employer
5. Parent, sibling, child, or other trusted family member
6. Close friend
7. Local or national news anchors
8. Celebrities, influencers, or public figures speaking about a disease that has touched them personally
9. Internet sites that provide health or medical information
10. People and places I follow on social media
11. Leaders from my faith-based institution
12. A national health organization (e.g. The American Cancer Society, etc.)
13. A government health organization, such as the CDC (Centers for Disease Control and Prevention) or state/local health department

If Q20a_1 (DOCTORS)= 1 or 2 OR Q20a_2 (Other medical professional) (DISAGREE) ASK:
Q20b. You mentioned you do not trust doctors and/or other medical professionals. What specifically causes you to feel this way? *Please be as specific as possible.* **(Open-end)**

SM1. Which of the following social media platforms do you use? *(Select all that apply)* **[RANDOMIZE]**

1. Instagram
2. Facebook
3. Twitter
4. TikTok
5. Snapchat
6. YouTube
7. Pinterest
8. Twitch
9. Reddit
10. WhatsApp
11. WeChat
12. Tumblr
13. Other (please specify ___) **[ANCHOR]**
14. I do not use social media **[ANCHOR, EXCLUSIVE]**

General Health Care Behavior

Now we'd like to know more about your health care behavior and perceptions in general.

S20a. Which of these would you say is your primary source for health care when it's not an emergency? *(Select one)*

1. Primary care physician
2. Primary care nurse practitioner or physician assistant
3. Urgent care
4. Emergency room
5. Clinic set in a pharmacy
6. Alternative medicine provider (e.g. acupuncturist, herbalist)
7. Specialist (e.g. cardiologist, physical therapist, chiropractor)
8. Other (please specify_________) **[ANCHOR]**

IF S20a<>1 ASK; IF S20a=1 AUTO-POPULATE S20a=1:

S20b. Do you have a primary care doctor? *(Select one)*

1. Yes
2. No

S21a. When seeing a health care provider, which statement regarding **gender** is true for you? *(Select one)* **[RANDOMIZE]**

1. I prefer to see a **male** health care provider
2. I prefer to see a **female** health care provider
3. The gender of the health care provider **does not matter to me [ANCHOR]**

S21b. When seeing a health care provider, which statement regarding **race** is true for you? *(Select one)* **[RANDOMIZE]**

1. I prefer to see a health care provider that is the **same race** as me
2. The race of the health care provider **does not matter to me**

S25b. Through which of the following ways do you communicate with or receive health-related information from health care providers? *(Select all that apply)* **[RANDOMIZE]**

1. Patient health portal (website or app where you can log in to access test results, prescription information, receive messages from your doctor, etc.)
2. Text Message (typically used for appointment reminders, prescription refill reminders, etc.)
3. Email
4. None of these **[ANCHOR]**

Q1aa. Thinking about your general health attitudes and habits, how much do you agree or disagree with each of the following statements? Please use a scale of 1 to 5 where 1 means, "Strongly Disagree" and 5 means, "Strongly Agree". **[RANDOMIZE]**

Strongly Disagree	Somewhat Disagree	Neither Agree nor Disagree	Somewhat Agree	Strongly Agree
1	2	3	4	5

1. I visit my doctor's office for regular check-ups, screenings, and wellness care, whether I'm sick or not
2. I get a physical each year
3. I only visit the doctor's office when I am sick
4. I avoid going to the doctor as much as possible
5. I consider myself healthy
6. I care a great deal about maintaining my health
7. I know what I need to do to stay healthy

Q1b. Thinking about racism and racial bias in health care, how does it affect your decision to seek out medical care? *(Select one)*

1. Makes me **extremely hesitant** to seek out medical care
2. Makes me a **little hesitant** to seek out medical care
3. It has **no impact** on my decision to seek out medical care

Connection to Cancer

We're nearing the end of our survey. The next few questions may be somewhat sensitive, but we would very much appreciate your feedback.

Programming Note: Show C21a and C21b on same screen

C21a. Have you ever been diagnosed with cancer?

1. Yes
2. No

If C21a=1 ASK:

C21b. What type(s) of cancer were you diagnosed with? *(Select all that apply)*

1. Breast
3. Cervical
2. Colorectal (Colon)
4. Leukemia
5. Lung
6. Ovarian
7. Prostate
8. Skin
9. Other (please specify ______)

Programming Note: Show C22a and C22b on same screen

C22a. Do you have a family history of **ANY** type of cancer?

1. Yes
2. No

If C22a=1 ASK:

C22b. What type(s) of cancer do you have a family history of? *(Select all that apply)*

1. Breast

2. Cervical
3. Colorectal (Colon)
4. Leukemia
5. Lung
6. Ovarian
7. Prostate
8. Skin
9. Other (please specify ___)

C26. To your knowledge, which of the following apply to any of your family members? *(Select all that apply)* **[RANDOMIZE]**

1. Have been told by a doctor that they have colon cancer
2. Have been told by a doctor that they have polyps in their colon or rectum
3. Have been told that they have a higher risk for colon cancer
4. Have been told to undergo genetic testing for colon cancer
5. Have been tested for colon cancer
6. Have discussed the results from their colon cancer tests with you **[ANCHOR AFTER CODE 5]**
7. None of these **[ANCHOR]**

IF C22b=3 or C26=1 ASK:
C26b. Given you've had family members who had colon cancer, please explain how important you think it is for you to get screened yourself. (Open-end)

C24a. Do you have any close friends that have had colon cancer?

1. Yes
2. No

Demographics/Lifestyles

We have just a few more questions.

S9. Are you currently covered by any kind of health insurance?

1. Yes
2. No

If S9=1 ASK:
S11. To which type of health care insurance plan do you belong? *(Select all that apply)* **[RANDOMIZE]**

1. Private insurance through employer. (Your employer pays for some or all of your coverage)
2. Private insurance that you pay for yourself
3. Medicare - A national insurance program run by the U.S. government, that provides access to medical care for Americans ages 65 and older, and to younger people who quality for Medicare, including those with disabilities and those with permanent kidney failure
4. State Insurance program (i.e. Medicaid, CHIP, FamilyCare, Medi-Cal, MassHealth) – Health insurance provided through your state for people with lower incomes or people with disabilities. This insurance supports medical and health-related services, such as doctor's visits, prescriptions, preventive care, disability benefits, and long-term nursing home services
5. Access to health care through military, VA or Tricare
6. Insurance through federal, state, or local employee plan
7. Other (please specify) **[ANCHOR]**
8. Not sure **[ANCHOR]**

If S11=2 ASK:

S11a. Did you buy your insurance through a private company (i.e. directly from the insurance company) or did you get it through a health care exchange marketplace such as health care.gov? *(Select one)*

1. Directly from insurance company
2. Through state or federal health care exchange, such as the health.gov marketplace

D2. What is the highest level of education you have completed? *(Select one)*

1. Some high school
2. High school diploma or equivalent
3. Trade or vocational training
4. Attending/attended some college
5. Associate degree
6. Bachelor's degree
7. Graduate degree (Master's)
8. Postgraduate degree (Doctorate)

D3. What is your employment status? Are you… *(Select one)*

1. Employed Full-time
2. Employed Part-time
3. Self-employed
4. Not Employed
5. Student
6. Disabled
7. Retired

S8. Which of the following best describes your <u>current annual household income</u>? Your best guess is fine. Please be reassured that all of your responses will remain completely confidential. *(Select one)*

1. Less than $12,000
2. $12,000 to $25,999
3. $26,000 to $39,999
4. $40,000 to $59,999
5. $60,000 to $79,999
6. $80,000 to $99,999
7. $100,000 - $149,999
8. $150,000 or more
9. Prefer not to disclose

D4. What is your marital status? Are you… *(Select one)*

1. Single / Never married
2. Married / Living with Partner
3. Separated / Divorced
4. Widowed
5. Prefer not to disclose

D5. Do you have children…? *(Select all that apply)*

1. I do not have children **[EXCLUSIVE]**
2. Yes, ages 18 or older
3. Yes, under the age of 18

[Programming Note: Show D4 and D5 on same screen]

D6. Which of the following best describes where you live? *(Select one)*

1. Urban
2. Suburban
3. Rural
4. Don't know

D1a. To what extent do you consider yourself to be religious? *(Select one)*

1. Not religious
2. Moderately religious
3. Very religious

IF D1a=2 or 3 ASK:
D1b.Which religion do you belong to or identify yourself most closely to? *(Select one)* **[SHOW LIST IN ALPHABETICAL ORDER]**

1. Baptist
2. Catholic
3. Eastern Orthodox
4. Islamic
5. Jewish
6. Lutheran
7. Methodist
8. Mormon
9. Protestant
10. Other (please specify ____) **[ANCHOR]**
11. Prefer not to disclose **[ANCHOR]**

IF D1a=2 or 3 ASK:
D1c. Which of the following best describes how often you attend church? *(Select one)*

1. Several times a week
2. Once a week
3. 2-3 times a month
4. Once a month
5. Several times a year
6. Once a year
7. Less than once a year
8. Never

IF D1a=2 or 3 ASK:
D1d. Have you ever received health or health care related information in a faith based setting (e.g. at church, bible study, a church sanctioned event, etc.)?

1. Yes
2. No

IF D1d=1 ASK:
D1e. Please describe the type of information you have received and the type of faith based settings you were in? (Open-end)

IF D1a=2 or 3 ASK:
D1f. Which of the following describes how your faith/religious beliefs influence health care decisions you make?

1. Has **no influence** on health care decisions
2. Has **some influence** on health care decisions
3. Has a **great deal of influence** on health care decisions

IF D1f=2 or 3 ASK:
D1g. In what ways does your faith/religious beliefs influence your health care decisions? (Open-end)

PT20. Please select the choice that best fits for you. *(Select one)*

1. The survey was too long, I wanted to stop taking it
2. The survey was too long, I just started clicking through to get to the end
3. The length of the survey was fine

That completes our survey. Thank you for your time!
